# Allele-Biased Expression in Differentiating Human Neurons: Implications for Neuropsychiatric Disorders

**DOI:** 10.1371/journal.pone.0044017

**Published:** 2012-08-30

**Authors:** Mingyan Lin, Anastasia Hrabovsky, Erika Pedrosa, Tao Wang, Deyou Zheng, Herbert M. Lachman

**Affiliations:** 1 Department of Genetics, Albert Einstein College of Medicine, Bronx, New York, United States of America; 2 Department of Psychiatry and Behavioral Sciences, Albert Einstein College of Medicine, Bronx, New York, United States of America; 3 Department of Epidemiology & Population Health, Albert Einstein College of Medicine, Bronx, New York, United States of America; 4 Dominick Purpura Department of Neuroscience, Albert Einstein College of Medicine, Bronx, New York, United States of America; 5 Department of Neurology, Albert Einstein College of Medicine, Bronx, New York, United States of America; 6 Department of Medicine, Albert Einstein College of Medicine, Bronx, New York, United States of America; University of South Florida, United States of America

## Abstract

Stochastic processes and imprinting, along with genetic factors, lead to monoallelic or allele-biased gene expression. Stochastic monoallelic expression fine-tunes information processing in immune cells and the olfactory system, and imprinting plays an important role in development. Recent studies suggest that both stochastic events and imprinting may be more widespread than previously considered. We are interested in allele-biased gene expression occurring in the brain because parent-of-origin effects suggestive of imprinting appear to play a role in the transmission of schizophrenia (SZ) and autism spectrum disorders (ASD) in some families. In addition, allele-biased expression could help explain monozygotic (MZ) twin discordance and reduced penetrance. The ability to study allele-biased expression in human neurons has been transformed with the advent of induced pluripotent stem cell (iPSC) technology and next generation sequencing. Using transcriptome sequencing (RNA-Seq) we identified 801 genes in differentiating neurons that were expressed in an allele-biased manner. These included a number of putative SZ and ASD candidates, such as *A2BP1* (*RBFOX1*), *ERBB4, NLGN4X, NRG1, NRG3, NRXN1,* and *NLGN1*. Overall, there was a modest enrichment for SZ and ASD candidate genes among those that showed evidence for allele-biased expression (chi-square, p = 0.02). In addition to helping explain MZ twin discordance and reduced penetrance, the capacity to group many candidate genes affecting a variety of molecular and cellular pathways under a common regulatory process – allele-biased expression – could have therapeutic implications.

## Introduction

Stochastic and imprinted monoallelically expressed genes influence differentiation, development and cellular function. Imprinted genes are expressed in a parent-of-origin manner, whereas in stochastic monoallelic expression either the maternal or paternal allele is active in a given cell. Imprinted genes are marked during gametogenesis by differential methylation at CpG-rich islands and by chromatin modifications, and typically maintain their transcription-competent state after fertilization [1]–[3]. Some genes, however, acquire parent-of-origin imprints post-fertilization [4], [5]. Approximately 100 imprinted genes have been identified [4]. Whole genome expression studies expanded the family of parent-of-origin gene expression in the brain, although a subsequent re-analysis has shown that many imprinted gene calls were likely false positive findings and needed independent confirmation [6]–[8]. Imprinting plays a key role in some neuropsychiatric conditions, such as Prader-Willi Syndrome and Angelman Syndrome [9]. Parent-of-origin effects have also been observed in some families with schizophrenia (SZ), bipolar disorder (BD) and autism spectrum disorders (ASD) [10]–[20]. In addition, some investigators have suggested that genetic imprinting influences clinical phenotype, with an imbalance between the effects of paternally and maternally expressed genes in the developing brain resulting in an extreme paternal (ASD) or maternal (SZ) pattern of behavior [14], [21], [22].

Stochastic monoallelic expression is widespread in mammalian genomes. Examples include T-cell receptor and immunoglobulin genes, pheromone receptors, p120 catenin, odorant receptors, and the 5q31-linked *PCDH* family of protocadherins [23]–[26]. Recently, Gimelbrant et al. showed that random monoallelic expression affected nearly 10% of genes expressed in lymphoblasts, and similar to some imprinted genes, there was a degree of plasticity in that biallelic expression was observed in some clones [27].

Stochastic monoallelic expression in the brain could help explain some interesting epidemiological features of neuropsychiatric disorders, such as discordance in monozygotic (MZ) twins, where a range of ∼30–90% has been found in SZ, ASD and BD (see discussion) [28]–[31].

Two experimental tools have emerged that provide the means to evaluate the role of allele-biased expression in neuronal differentiation and neuropsychiatric disorders; iPSC technology, and next generation sequencing (RNA-Seq). We, along with other groups, are using iPSCs for *in vitro* disease modeling in a variety of neuropsychiatric disorders [32]–[37]. In addition to their utility for disease modeling in terms of identifying patient vs control differences in gene expression, morphology and neuronal function, iPSCs can also be used to study human neurogenesis, which is particularly relevant to SZ and ASD considering that both have a neurodevelopmental basis [38]–[40].

RNA-Seq provides increased sensitivity and the capacity to detect novel transcripts [41]–[44]. It is also an ideal platform to assess allele-biased expression by quantifying differences in expression that might occur across heterozygous single nucleotide polymorphisms (SNPs) [45]. We have used this approach to screen for allele-biased expression in differentiating human neurons. The findings highlight the degree to which allele-biased expression occurs during human neurogenesis and suggest a plausible mechanism to explain incomplete penetrance and MZ twin discordance in SZ, ASD, and other neuropsychiatric disorders.

## Materials and Methods

### iPSC Development and Neuronal Differentiation

iPSCs were grown and induced to differentiate into neurons using two different protocols: A and B, which are described in detail in Methods S1. Briefly, in protocol A, iPSCs were maintained on irradiated mouse embryo fibroblasts supplemented with FGF2 (10 ng/ml). Colonies were subsequently detached and grown as embryoid bodies (EBs) on non-adherent plates in the absence of FGF2. After 4 days, EBs were plated on laminin, which resulted in the development of clusters of neurons [37]. These were manually dissected after 10 days. The neurons derived from this protocol are primarily glutatmatergic (∼90%) [37]. In protocol B, iPSCs were maintained on matrigel and mTeSR1^®^ (StemCell Technologies, Vancouver, Canada). EBs were formed and neural rosettes were cultivated using standard techniques [34], [36]. Neurons emerged after rosettes were isolated and grown on Poly-dL-Ornithine/laminin coated plates. Neurons (generally an equal mix of glutamatergic and GABAergic neuorns) were harvested after 14 days.

RNA-Seq was carried out using line iPSC-1, which was derived from a control female. Validation by Sanger sequencing was carried out on iPSC-2 (a control male), and 3 lines derived from male subjects with SZ (SZ39, SZ97 and iPSC-15, the latter of which has a 22q11.2 deletion).

### RNA-Seq

Paired-end RNA-Seq was carried out using an Illumina HiSeq 2000 instrument, as described in Methods S1 and in our previously published work (GEO accession number GSE32625) [36]. In the current study, we reprocessed the previously reported RNA-Seq data to identify allele-biased gene expression [36]. The read length was 104 bases for each of the paired-end reads. A summary of the data relevant to this study is shown in Table 1. Genomic DNA was analyzed using the Affymetrix Genome-Wide Human 6.0 array and genotypes were scored using the Affymetrix Power Tools (APT) software based on the Birdseed calling algorithm. A total of 905,625 SNPs were called (call rate was 98.98%), and the average rate of heterozygosity and homozygosity was 28.04% and 70.94%, respectively (253,585 heterozygous SNPs were identified). Parental DNA was also genotyped using the Affymetrix Genome-Wide Human 6.0 array (paternal call rate, 98.44%; maternal call rate 98.34%). To reduce the known bias in mapping RNA-Seq reads towards reference alleles, we aligned the raw RNA-Seq reads to a modified reference human genome (hg19) by the software bowtie 2 in which bases at the identified heterozygous sites were replaced with “N” [46]–[48]. Reads mapped to heterozygous sites were then analyzed. To reduce false positives, we focused on sites that were covered by at least 10 high-quality reads and excluded bases with a Phred score lower than 13. We used samtools/bcftools (mpileup command) to record high-quality reads mapped across each heterozygous SNP site. We then called the consensus base(s) (by maximizing the posterior probability given the read distribution and average base quality), and calculated the phred-scaled probability of the base being called a homozygote or heterozygote [49]. Subsequently, the numbers of reads for the reference and alternative alleles were counted and used for a binomial test to determine if the ratio of the two numbers significantly deviated from 0.5, reflecting the null hypothesis that both alleles were equally expressed. The resulting p-values were further adjusted by multiple-testing correction using the B-H method [50], [51]. Sites with an adjusted p-value (i.e., FDR) <0.05 and a phred-scaled probability ≥20 were considered to be expressed in an allele-biased manner. These analyses were carried out for iPSCs and differentiated neurons separately; the resulting allele-biased expressed SNP sites are listed in Table S1. Genes with allele-biased expression were those containing SNPs shown in Table S1 that mapped between transcription start and termination sites, having an expression value >1 FPKM.

**Table 1:** Summary of RNA-Seq and SNP array data.

	iPSCs	NEURONS
N of reads (pairs)	269,672,486	222,127,542
N of paired-match reads	121,620,928	115,280,074
N of single-match reads	39,276,866	33,721,936
Ratio of mapped fragments	74.30%	82.30%
Total SNP calls	905,625	905,625
Heterozygotes	253,585	253,585
Total SNPs (>/ = 1 read)	51,161	63,489
Total SNPs (>/ = 10 high quality reads)	8,358	13,576
Binomial (FDR adjusted p-value <0.05) and monoallelic consensus by samtools	543	1542
Number of genes (FPKM>1)	314	801

RNA-Seq reads for some genes showed a preference for one allele in iPSCs, with a switch to the other in differentiating neurons. Heterozygous SNPs showing >4-fold changes in allele preference during differentiation were tabulated (Table 2).

**Table 2:** Allele switching during transition from iPSCs to neurons.

chr	SNP ID	ref	alt	iPSC ref	iPSC alt	Neurons ref	Neurons alt	Genes
chr1	rs12123760	T	C	2	8	21	5	ST6GALNAC3
chr1	rs10492963	C	T	1	13	13	1	RERE
chr2	rs3811568	A	G	11	2	2	9	FMNL2
chr2	rs6757809	T	C	2	13	10	2	NOL10
chr2	rs4674015	A	G	1	15	10	2	SPATS2L
chr3	rs9860614	A	G	8	2	0	11	RUVBL1
chr3	rs9817055	G	A	16	1	0	16	CLASP2
chr3	rs1975760	T	C	2	16	13	3	ZNF148
chr3	rs3846072	C	T	1	9	11	1	MED12L/P2RY14
chr3	rs7644975	G	A	1	10	23	4	ERC2
chr4	rs7678728	T	G	1	9	10	2	ANK2
chr4	rs236985	G	A	2	8	9	2	AFF1
chr5	rs7712332	A	G	13	2	2	11	MSH3
chr5	rs2115436	T	A	2	10	13	2	SCAMP1
chr6	rs9373571	T	A	22	1	2	8	ASCC3
chr6	rs577372	A	G	2	9	11	2	MTO1
chr7	rs2691561	T	A	13	3	2	15	SNX13
chr7	rs258654	C	T	9	1	1	14	CACNA2D1
chr7	rs674462	A	T	2	9	9	2	MRPL32
chr8	rs6987331	T	G	12	3	1	9	SNTB1
chr8	rs2980683	G	C	1	10	10	1	AGPAT5
chr9	rs7019027	G	A	12	3	0	11	KDM4C
chr9	rs1411675	G	A	13	3	1	9	FXN
chr9	rs2297499	C	G	9	2	4	16	TLE4
chr9	rs7020390	A	G	2	9	10	2	UHRF2
chr10	rs1127047	G	A	9	1	2	8	PITRM1
chr10	rs1937971	A	C	17	0	3	16	NRG3
chr10	rs11817793	G	A	8	2	1	10	LARP4B
chr10	rs2418929	C	G	12	2	1	10	KIF20B
chr10	rs10795321	C	G	0	13	32	5	FAM188A
chr10	rs7084542	A	G	3	14	39	9	RP11-429G19.2
chr12	rs4130296	A	C	3	15	22	3	FBXW8
chr13	rs11619378	T	G	12	2	2	16	ABHD13
chr13	rs7983071	G	A	10	2	1	20	GPC6
chr13	rs9535499	G	C	3	34	21	4	DLEU7/DLEU7-AS1
chr14	rs12893288	C	T	16	4	3	13	intergenetic
chr14	rs10131048	T	C	3	14	15	3	ZFYVE26
chr16	rs17139246	T	C	10	2	0	14	RBFOX1
chr17	rs2106663	G	A	2	10	9	1	BRIP1
chr17	rs2302235	G	A	4	21	9	2	FAM20A
chr17	rs9646411	C	T	0	10	10	0	AMAC1L3
chr18	rs7233697	T	C	8	2	1	17	MEP1B

Genes that showed >4 changes in allele distribution occurring during the transition from iPSCs to neurons. Abbreviations: chromosome number (chr); SNP identification number (SNP ID); reference allele (ref); alternative allele (alt); high quality reads in iPSCs (iPSC ref and iPSC alt); high quality reads in neurons (Neuron ref and Neurons alt).

We also assessed potential sequencing chemistry and mapping bias for allele-biased SNP calls. There was no preference towards any one of the four nucleotides (Figure S1).

Selected monoallelic SNPs were validated by capillary sequencing using RT-PCR amplified material (see Methods S1).

## Results

RNA-Seq was carried out on iPSCs and differentiating neurons harvested from iPSC-1 using procedure A, which leads primarily to the production of glutamatergic neurons [37]. Of the 253,585 heterozygous SNPs subjected to analysis for allele-biased expression, a total of 1,909 satisfied the criteria described in the methods section (Table S1). These mapped to a total of 314 genes in iPSCs and 801 in differentiating neurons (Tables S2 and S3, respectively). These included 181 neuronal genes that contain two or more highly significant allele-biased SNPs, of which several are candidate genes for SZ, ASD, and intellectual disability (see below). Based on genotyping data obtained from the parents of iPSC-1, the ratio of paternal to maternal allele-biased SNPs is 1.2∶1 in iPSCs and 1.1∶1 in neurons, excluding the X-chromosome, indicating that there was no significant parent-of-origin bias.

Overall, 24.5% of all genes expressed in an allele-biased manner in iPSCs and 12.4% in neurons were X-linked. The abundance of X-linked genes is consistent with the finding that human iPSCs often maintain a clonal X-chromosome active state [52]. However, in other studies the pattern of X-chromosome inactivation has been found to be heterogeneous [53], [54]. All of the X-linked monoallelic SNPs were maternal, with the exception of *XIST*, which is expressed on the inactive X-chromosome (paternal in this sample), and *WASF4P* and *KIF4*, suggesting that the strict criteria used for our definition of allele-biased expressed SNPs resulted in few false positives.

### Validation of X-linked and Imprinted Genes by Sanger Sequencing

Technical factors can inflate the false positive rate when RNA-Seq data are used to evaluate allele-biased expression; consequently, we validated several genes using Sanger dideoxy sequencing [47]. We also analyzed iPSCs and neurons that were maintained and induced to differentiate using a different protocol to determine whether culture conditions might influence allele-biased expression (see Methods S1). Finally, when SNPs were informative, we validated allele-biased expression in other iPSC lines.

**Figure 1:**
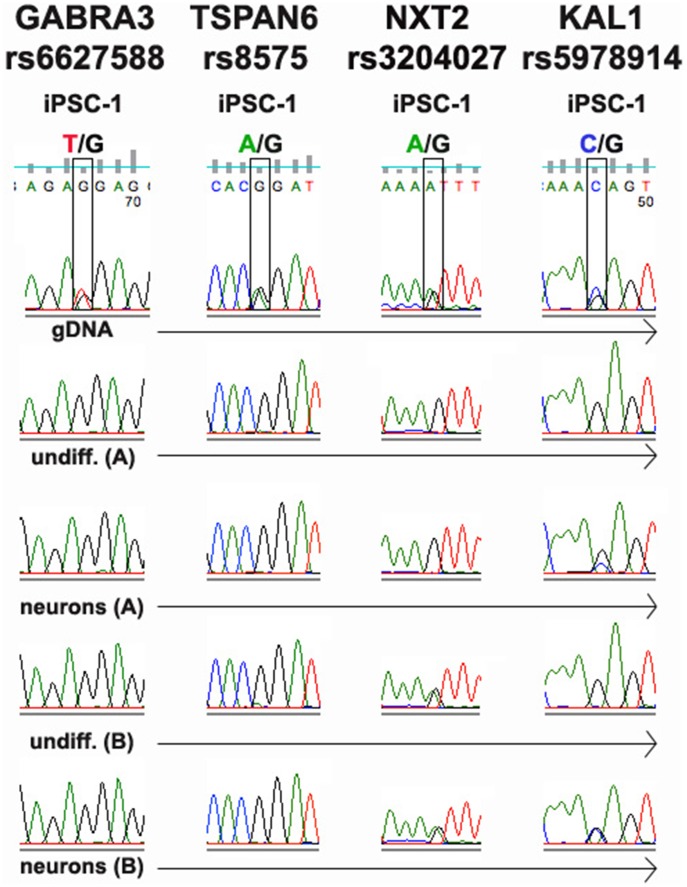
Sanger sequencing of selected X-linked genes. Top panel in each strip is sequence of genomic DNA (gDNA) for iPSC-1, a control subject, confirming heterozygosity of each SNP. Other strips are sequences of cDNA derived from undifferentiated (undiff) and neurons cultivated under growth and differentiation conditions A and B (see Methods S1 for details). The RNA samples used to generate the Sanger sequencing data for iPSC-1 protocol A (undifferentiated iPSCs and neurons) were the same samples used for RNA-Seq.

First, we analyzed four X-linked genes (*GABRA3, TSPAN6, NXT2,* and *KAL1*). Heterozygosity for each was confirmed by sequencing gDNA (Figure 1). However, only one peak was detected when cDNA was sequenced from template RNA derived from iPSCs and neurons derived from the line iPSC-1 grown using protocol A, which was used in the RNA-Seq experiment. An exception is *KAL1* in neurons, which showed allele-bias rather than complete monoallelic expression.

Each of the X-linked validated SNPs met the criteria described in the methods section except for rs6627588 in *GABRA3.* The expression of only a single allele shows that our conservative method for determining allele-biased expression is leading to some degree of type II error.

Interestingly, a somewhat different pattern was observed using iPSCs and neurons grown under condition B. Here there is also clear evidence for monoallelic expression in *GABRA3* and *TSPAN6* in both iPSCs and neurons, and in iPSCs for *KAL1*. However, biallelic expression is seen for *KAL1* in neurons and for *NXT2* in iPSCs and neurons, suggesting that X-activation/inactivation is somewhat variable under different culture/differentiation conditions.

X-linked markers could not be verified in the other iPSC lines since they were each derived from male subjects.

**Figure 2:**
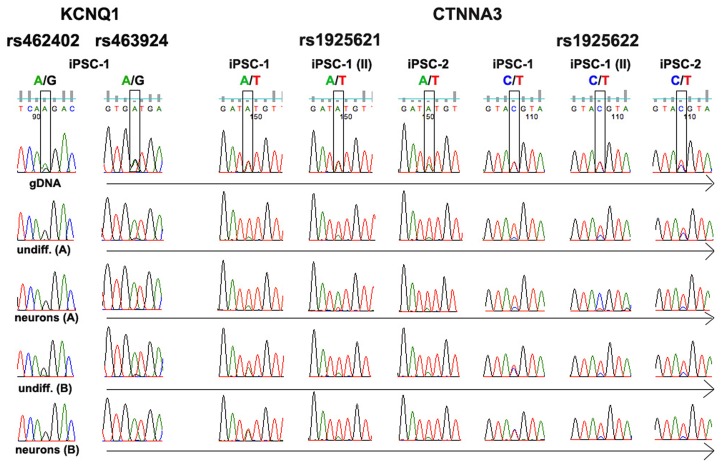
Imprinted genes. SNPs in *KCNQ1* and *CTNNA3* were validated by Sanger sequencing. See legend Figure 1 for details. iPSC-1 (II) is a biological replicate of iPSC-1.

We also analyzed *CTNNA3*, which has been shown to be imprinted in placenta, and *KCNQ1*, a known imprinted gene [3], [55], [56]. *KCNQ1* was not included in the list shown in Tables S2 and S3 because the minimum FPKM >1 threshold was not achieved for this gene. Nevertheless, Sanger sequencing clearly shows allele-biased expression for both genes (Figure 2). Thus, similar to the expression pattern for *GABRA3,* this further illustrates that our allele-biased algorithm is associated with some type II error.

Two informative SNPs were analyzed in *CTNNA3*, rs1925621 and rs1925622. As seen in Figure 2, a single allele at rs1925621 is expressed in undifferentiated iPSCs and neurons derived from iPSC-1 grown using protocol A. However, when the cells were cultivated under protocol B, expression was essentially biallelic for both SNPs in iPSC-1. Interestingly, when a biological replicate of iPSC-1 was analyzed (iPSC-1 (II)), the other parental allele was activated in neurons grown using protocol A. In addition, in in the replicate sample, allele-bias was also seen in neurons grown using protocol B. Similarly, when these SNPs were analyzed in another iPSC line (iPSC-2, which was derived from another control subject), allele-biased expression was seen in iPSCs and neurons cultivated under both protocols.

The same results were obtained when rs1925622 was analyzed.

These data confirm allele-biased expression for *CTNNA3* under some culture and differentiation conditions, and suggest that the phenomenon is random in this system and not imprinted.

### Validation of SZ and ASD Candidate Genes

Considering the parent-of-origin effects seen in some families and the possibility that allele-biased expression could help explain MZ twin discordance and reduced penetrance, we were interested in specifically validating SZ and ASD candidate genes that showed evidence for allelic imbalance in the RNA-Seq experiment. Overall, there were 48 SZ candidate genes and 26 ASD candidates expressed in an allele-biased manner in neurons, while in iPSCs, there were 17 and 9, respectively (downloaded from Allen Brain Atlas list of 883 SZ and 244 ASD candidate genes) (Table S4). A Chi-square test was used to determine whether a statistically significant enrichment of SZ/ASD genes is found in the allele-biased gene sets. More specifically, a 2×2 table was set up to compare the allelic expression status (biased vs non-biased) of SZ/ASD candidates vs. all others; genes in both groups were selected for containing at least one SNP covered by >/ = 10 reads. A significant enrichment was found for SZ/ASD candidate genes expressed in neurons (p = 0.02), while no significant enrichment was observed in iPSCs (p = 0.14).

The SZ and ASD genes that showed the most robust evidence for allele-biased expression, based on containing more than one significant SNP, were *RBFOX1 (A2BP1)* (15 SNPs), *NLGN4X* (14 SNPs), *NRG3* (10 SNPs), *NRG1* (9 SNPs), *CASK* (7 SNPs), and *ERBB4, CNTNAP2, NLGN1,* and *NPAS3*, each of which had 3 SNPs. Other genes of interest, such as *NRXN1* and *DISC1,* were only represented by one SNP.

**Figure 3:**
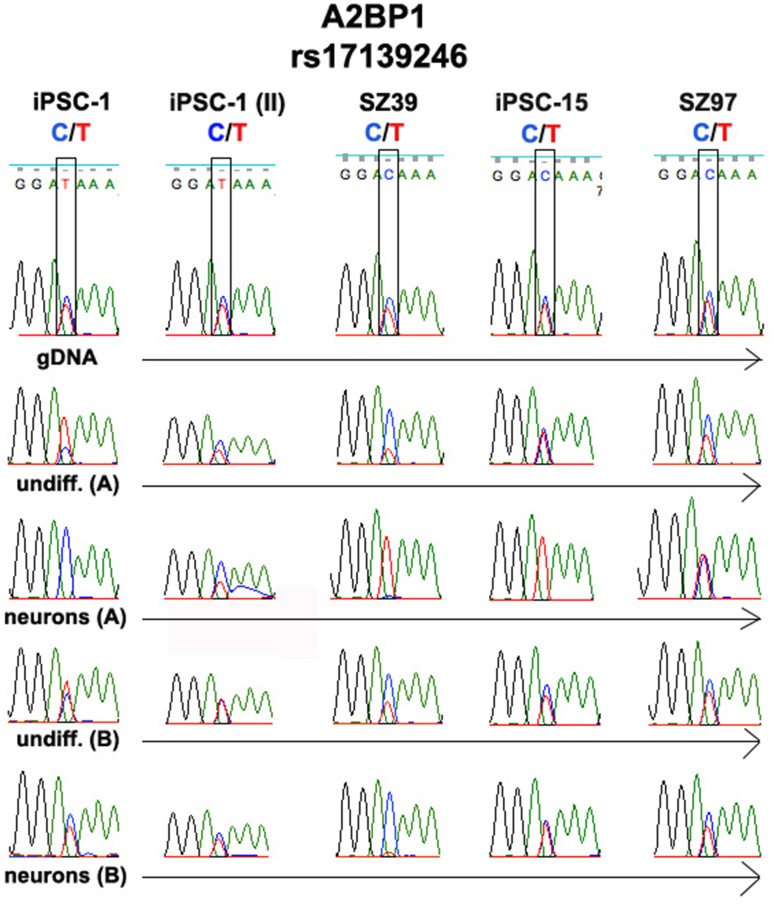
Validation of *A2BP1*. SZ39, SZ97 and iPSC-15 are iPSC lines developed using fibroblasts from patients with SZ.

We validated allele-biased expression in five SZ and ASP candidate genes, including *A2BP1* (Figure 3). RNA-Seq data showed that in iPSC-1, one parental allele was expressed at this locus in undifferentiated iPSCs (T allele), and the other (C allele) in neurons (Table S1; compare columns H, I, N and O for rs17139246). This was one of 42 genes that showed evidence for a switch from one parental allele to the other during the transition from iPSCs to neurons (Table 2). The switch in *A2BP1* alleles was confirmed by Sanger sequencing (Figure 3; undifferentiated iPSCs and neurons grown under protocol A, which was produced from the same sample used for RNA-Seq). However, when grown under protocol B, expression is essentially biallelic (there is a hint of allele-biased expression, but this should be viewed cautiously because capillary sequencing is only semi-quantitative).

Switching from one allele to the other could either reflect random activation of parental alleles occurring independently in iPSCs and neurons, or programmed expression of one parental allele in iPSCs and the other in neurons. Random expression is supported in the analysis of a biological replicate; iPSC-1 (II). As seen in Figure 3, the expression pattern was different compared to the original sample; some degree of allele-biased expression seen only for the “C” allele in iPSCs and neurons grown under protocol A suggesting that allele-biased expression in *A2BP1* during early neurogenesis is random. Whether other genes shown in Table 2 switch allelic preference during differentiation by the same random mechanism is under investigation.

Further validation of allele-biased expression in *A2BP1* is seen in three other iPSC lines, each of which is heterozygous at rs17139246. Allelic bias and a switch were clearly seen in SZ39 with protocol A. With protocol B however the predominate allele was C, as opposed to T in neurons grown under protocol A. These findings support the idea that allele-biased expression of *A2BP1* is a stochastic process.

For the other two lines, iPSC-15 showed allele-biased expression protocol A neurons, but not in undifferentiated cells, while in SZ97, expression was essentially biallelic.

**Figure 4:**
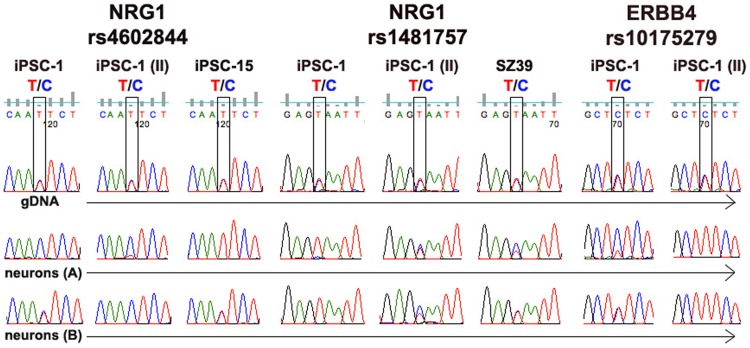
Validation of *NRG1* and *ERBB4*. The *NRG1* SNPs rs4602844 and rs1481757 map near the promoter of the NRG1 isoform HRG-β1C, and an intron in the GGF2/HRG-β1D isoforms, respectively.

Validation of *NRG1* and *ERBB4* is shown in Figure 4. For *NRG1*, two SNPs were analyzed - rs4602844 and rs1481757, which map near the promoter of the NRG1 isoform HRG-β1C and an intron in the GGF2/HRG-β1D isoforms, respectively. Sanger sequencing confirmed the RNA-Seq allele-biased calls in iPSC-1 neurons for rs4602844 (expression was too low in undifferentiated cells for reliable sequencing). In addition, we were able to confirm allele-biased expression for this SNP in the replicate sample, iPSC-1 (II), and in iPSC-15. With protocol B, a different pattern emerged. Neurons derived from both iPSC-1and iPSC-15 showed biallelic expression, but in iPSC-1 (II), allele bias was clearly seen. For rs1481757, allele-biased expression was seen in iPSC-1 but not in iPSC-1 (II), for both protocol A and B neurons. For SZ39, there was a suggestion of allele-biased expression only in protocol A.

These findings suggest that isoforms within the same gene may be subject to different regulatory signals with respect to allele-biased expression.

For rs10175279 (*ERBB4*) allele-biased expression of the C allele was seen in iPSC-1 in protocol A. However, in iPSC-1 (II), the other parental allele (T) is expressed. Furthermore, while biallelic expression was detected in iPSC-1 in protocol B, expression was biased towards the T allele in the replicate. These findings suggest that similar to *A2BP1*, allele-biased expression in *ERBB4* is random.

Finally, Sanger sequencing was also carried out for SNPs in *NRG3* and *AUTS2.* This analysis essentially showed a similar mixed pattern of allele-biased or biallelic expression for neurons that varied according to growth and differentiation protocol (Figure S2).

To summarize, the validation of X-linked, imprinted, and SZ and ASD candidate genes confirms the monoallelic SNP calls made in the RNA-Seq analysis and demonstrates that the phenomenon is not restricted to a single iPSC line. In addition, allele-biased expression is random for some genes, and can vary depending on culture conditions. These findings are similar to the change that occurs in NANOG mRNA (from a monoallelic to biallelic pattern of expression) in ES cells treated under different culture conditions, as well observations made on allele-biased gene expression in lymphoblasts [27], [57].

## Discussion

**Figure 5:**
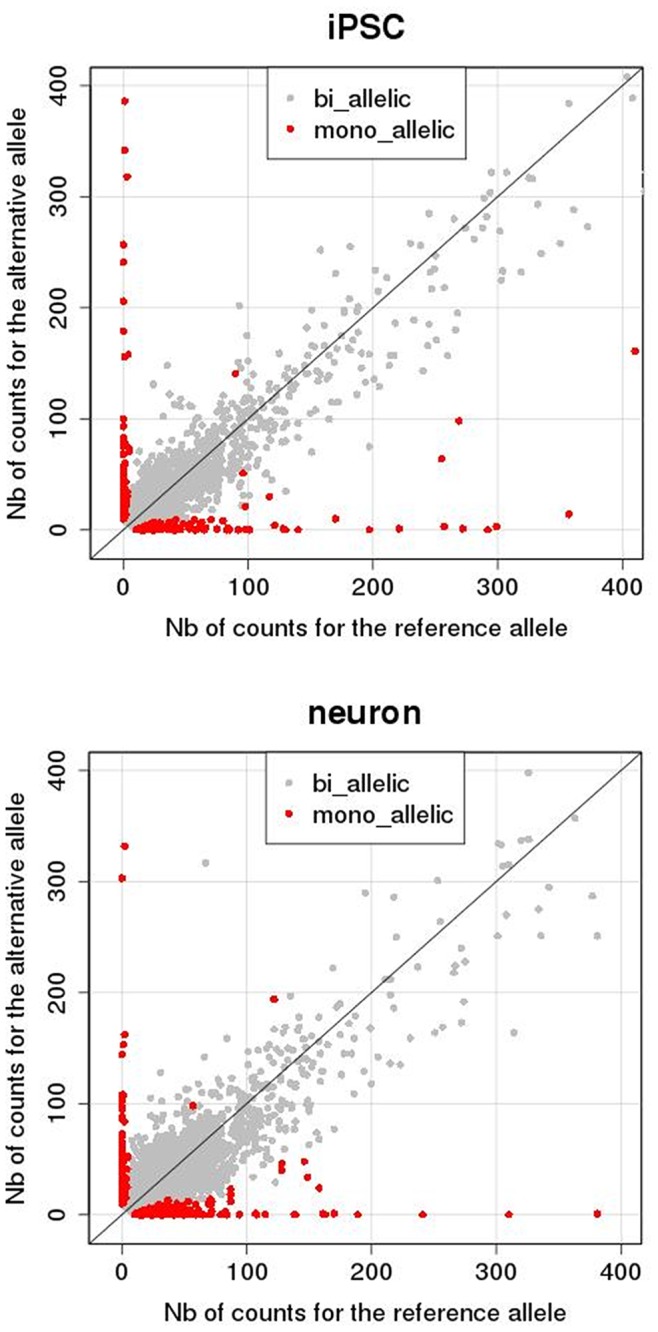
Reference vs alternative RNA-Seq reads at heterozygous SNPs. Y-axis, number (Nb) of reads with the alternative allele (211 in iPSCs, 619 in neurons); X-axis, number of reads with the reference allele (332 in iPSCs, 923 in neurons). Biallelic (grey); allele-biased expression (black).

Using RNA-Seq to identify allele-biased expression genome-wide has the capacity to add a novel dimension to our understanding of transcriptional regulation in different cell types. However, interpreting RNA-Seq data is complicated and can be confounded by false positive allele-biased calls [47]. One of the more important factors is *in silico* mapping bias when RNA-Seq reads are aligned to the reference genome, as described in the methods section. In this study, the reference allele was called with a 1.5∶1 bias (Figure 5), despite precautions taken while aligning RNA-Seq reads (see methods). The cause of this persistent bias is under investigation, but does not appear to arise from sequencing bias (Figure S1). Nevertheless, the validation studies strongly support the RNA-Seq findings.

Allele-biased expression could be caused by epigenetic phenomena or a genetic process, such as copy loss or promoter variants. It has been suggested that most allele-biased expression is due to genetic factors [47], [48]. This may well be the case for many of the genes in this study. However, several of the genes we validated, including *CTNNA3* and the SZ candidate genes *A2BP1* and *ERBB4,* showed random allele-biased expression suggesting an epigenetic phenomenon. Distinguishing between genetic and epigenetic processes (both random monoallelic expression and imprinting) will require a much more extensive analysis using several different lines and multiple replicates. This is an important consideration in SZ and ASD because an epigenetic process accounting for allele-biased expression could have an impact on determining the efficacy of epigenetics-based treatment. Confirmation in differentiating mouse and/or human neurons derived from embryonic neural progenitors is also needed.

Finally, allele-biased expression, in particular a stochastic model, has the potential to explain the ∼50% MZ twin discordance seen in SZ [28]–[31]. Here, a disease-causing mutant allele would have an equal probability of being expressed (causing disease), or suppressed (avoiding disease) if such a process occurred in a pathway or cortical segment that was clonally derived. A stochastic model is also consistent with the observation that the prevalence of SZ in the children of discordant and concordant MZ twins is similar [28].

GWAS and CNV discovery have resulted in the identification of numerous candidate genes for neuropsychiatric disorders, a number that is bound to increase dramatically with rare variants detected using exome and whole genome sequencing. Translating the heterogeneous mix of susceptibility genes into novel medications could prove to be very challenging. This study presents the possibility of incorporating seemingly disparate candidate genes into a common molecular pathway – epigenetic regulation leading to allele-biased gene expression. Based on this idea, it is conceivable that one approach to treating a subgroup of patients would be at the epigenetic level (assuming a non-genetic cause of allele-biased expression) using drugs capable of activating a dormant normal allele as a means to buffer the effects of their monoallelically expressed abnormal partner. Proof of principle for this concept has recently been demonstrated in an animal model of Angelman Syndrome [58].

## Supporting Information

Figure S1 **Distribution of nucleotides for allele-biased SNPs.**
(JPG)

Figure S2 **validation of replicates and additional genes.**
(JPG)

Table S1 **Heterozygous SNPs showing allele-biased expression.**
(XLS)

Table S2 **allele-biased genes in undifferentiated iPSCs.**
(XLS)

Table S3 **allele-biased genes in neurons.**
(XLS)

Table S4 **SZ and ASD genes showing allele-biased expression in iPSCs and neurons (from Allen Brain Atlas list of 883 SZ and 244 ASD candidate genes).**
(XLSX)

Methods S1 **Detailed methods, reagents and PCR primers.**
(DOC)
