# Supplementary figures and images for: Allele-Biased Expression in Differentiating Human Neurons: Implications for Neuropsychiatric Disorders
Source: PLoS One. 2012 Aug 30;7(8):e44017. doi: 10.1371/journal.pone.0044017 (PMC3431331; doi:10.1371/journal.pone.0044017)

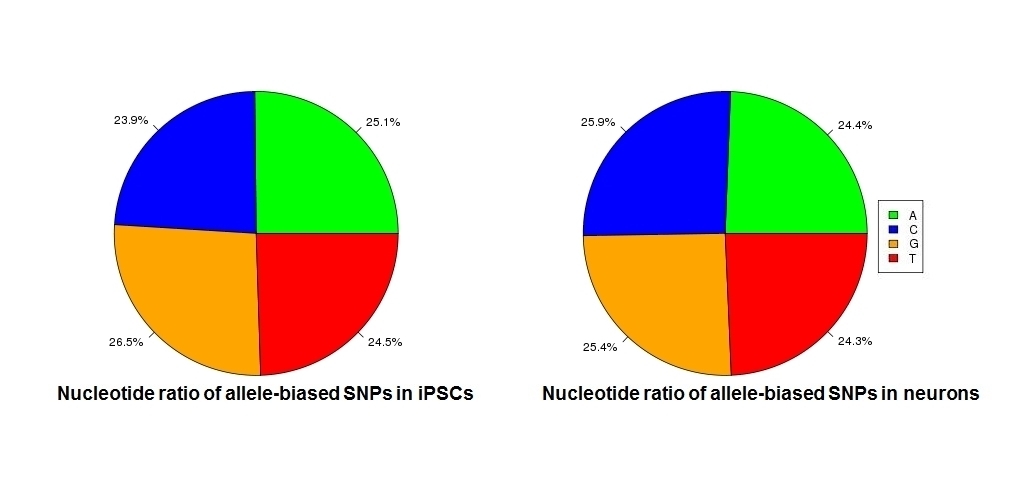

Supplement: Figure S1 — Distribution of nucleotides for allele-biased SNPs. (JPG) [file pone.0044017.s001.jpg]

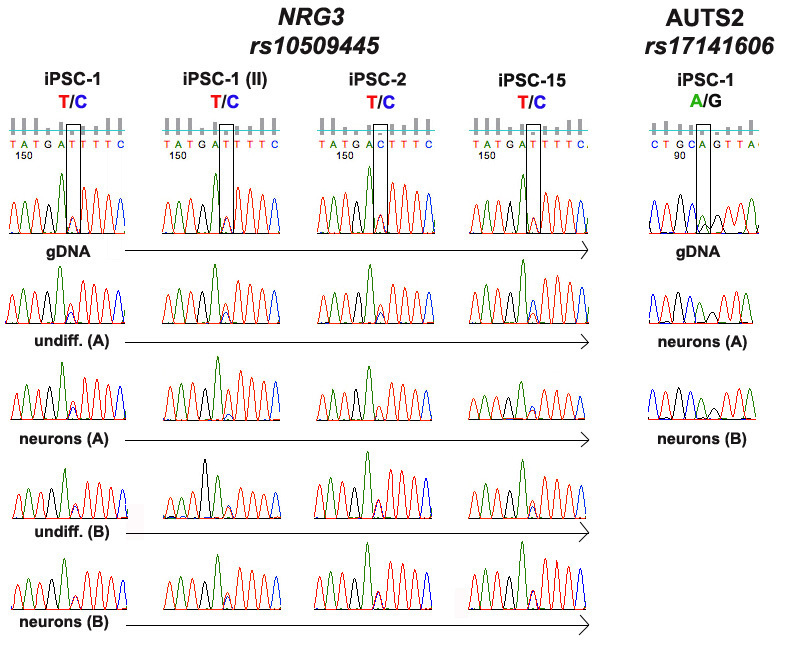

Supplement: Figure S2 — validation of replicates and additional genes. (JPG) [file pone.0044017.s002.jpg]
