# Supplementary material for: Allele-Biased Expression in Differentiating Human Neurons: Implications for Neuropsychiatric Disorders
Source: PLoS One. 2012 Aug 30;7(8):e44017. doi: 10.1371/journal.pone.0044017 (PMC3431331; doi:10.1371/journal.pone.0044017)
Supplement: Methods S1 — Detailed methods, reagents and PCR primers. (DOC) [file pone.0044017.s007.doc]

**Materials and methods**

**Human Subjects**

IPSC lines were generated from fibroblasts obtained from two healthy controls (29 year old female (iPSC-1) and a 59 year old male (iPSC-2). In addition, lines were generated from a 31 year old patient with 22q11 del diagnosed with SZ (iPSC-15), and two patients with SZ obtained from the Coriell cell repository (Camden, NJ); GM02039, a 22 year old male with an age of onset at age 6 and no other family history (SZ39); and GM02497, a 23 year old male with age of onset at 15 and family loading for SZ (affected father and 3 siblings) (SZ97). Skin biopsy was the source of fibroblasts for all subjects except SZ39, which was developed from liver fibroblasts. All subjects were Caucasian. The study was approved by the Albert Einstein College of Medicine committee on clinical investigation.

**Generating iPSC Lines**

Skin biopsy samples were transferred to a small Petri dish containing 2-3 ml Skin Fibroblast Media (SFM) consisting of RPMI 1640, 10% FBS, 1% pen/strep, 10ng/ml FGF2. The sample was incubated at room temperature for 15 minutes. Medium was carefully aspirated and replaced with 1-2 ml of collagenase type II solution (3mg/ml collagenase II dissolved in DMEM High Glucose [Worthington Biochemical Corp. Lakewood, NJ: GIBCO/Invitrogen, Carlsbad, CA]). The tissue was chopped into small pieces using 2 sterile scalpels, after which they were allowed to incubate at 37^o^C for 1­2 hours depending on size. The sample was then collected in a 15ml falcon tube and washed with SFM (serum free medium). Tissue was collected by centrifugation at 1200 rpm for 4 minutes. The pelleted sample was then suspended in SFM and plated in a T12.5 ml flask at 37^o^C in 5% CO_2_ for 3 days without changing medium or manipulation, to allow fibroblasts to adhere. Then cells were subsequently fed every 2 days with RPMI 1640 containing 10% FBS until a confluent culture was obtained (~3 weeks). The cells were reprogrammed into iPSCs using the Yamanaka transcription factors (c-MYC, SOX2, Klf4, and OCT4 [POU5F1]) driven by a retroviral LTR as previously described (1). The passage numbers for the experiments carried out in this study were: IPSC-1, passages 5 (protocol A) and 19 (protocol B); SZ39, passages 16 (protocol A) and 17 (protocol B); iPSC-15, passages 11 (protocol A) and 24 (protocol B). For SZ97 protocol A, passage 8 was used for iPSCs and passage 21 for neurons. For protocol B, passage 10 was used. A complete description of the lines used in this study, with respect to validating iPSC phenotype, capacity to differentiate into all three germ layers and karyotype have previously been published (2, 3)

**Culture of Human iPSCs (Protocol A)**

Stem cell lines were maintained on irradiated mouse embryonic fibroblasts (MEFs) in hES medium consisting of knockout (KO)-DMEM, 1mM pen/strep, 1 mM Gluta-MAX, 1X nonessential amino acids (NEAA), 55 μM β-mercaptoethanol (β-ME) (Gibco, Carlsbad, CA), 10% knockout (KO) serum replacement (Invitrogen, Carlsbad, CA), 10% plasmanate (Talecris Biotherapeutics, Research Triangle Park, NC), and 10 ng/ml FGF2 (R&D Systems Inc., Minneapolis, MN). For maintenance of undifferentiated colonies, differentiated cells were manually removed and undifferentiated cells were passaged once a week. Karyotyping was carried out by Cell Line Genetics (Madison WI). All lines exhibited a normal (46XX and 46XY) karyotype, and the one derived from a patient with VCFS showed a single copy of 22q11.2 based on FISH analysis using a TUPLE1 probe.

**Neuronal Differentiation (Protocol A)**

Neuronal differentiation was induced in the following manner. IPSCs were treated with 1mg/ml dispase and detached using a cell scraper. Colonies were pooled together and washed in hES medium and grown as cellular aggregates (embryoid bodies; EBs) on Ultra Low Adherence 6-well plates (Costar catalog # 3471, Corning, Corning, NY) without FGF2 (day 0). EBs were maintained with a daily medium change for 4 days. Medium was changed by allowing the aggregates to settle by gravity, followed by removal of the supernatant and addition of fresh, pre-warmed medium. On day 4, aggregates were switched to a neural induction medium consisting of DMEM/F12, 1X N2 supplement (Invitrogen, Carlsbad, CA), 0.1mM NEAA, 2μg/ml heparin (Sigma-Aldrich Corp., St. Louis, MO), again omitting FGF2. Cultures were fed every other day by allowing aggregates to settle by gravity, removing supernatant, and replacing with fresh medium. On day 6, the EBs were transferred to laminin coated plates (20μg/ml [Roche]) to which they adhered. Feeding was carried out in neural induction medium every other day. Within 1-2 days, clusters of differentiated cells, including neurons, began to appear. Neurons were harvested on day 10. More than 90% of the cells are glutamatergic based on VGLUT2 staining, microarray and RNA-Seq findings (2, 3).

**Culture of Human iPSCs (Protocol B)**

iPSCs were maintained in mTeSR1 medium (Stem Cell Technologies) for approximately 5-6 days. Colonies were checked for spontaneous differentiation under a dissecting microscope and were removed manually. The medium was then changed to N2 (DMEM/F12, 1X N2; Invitrogen. This was designated as day 0. The following day (Day 1), medium was changed to N2 plus 1μM Dorsomorphin (CALBIOCHEM). On day 2, embryoid bodies (EBs) were created. Briefly, iPSCs were checked again for spontaneous differentiation and fresh N2 medium plus 1μM Dorsomorphin was added. Colonies were cut with a 5ml glass serological pipet using wide strokes to generate large fragments. A cell scraper was then used to detach remaining cells. EBs were aliquoted to a 6 well ultra-low attachment plate (Corning). On day 4, EBs were collected in a 15ml tube and allowed to settle via gravity for 5 minutes. Supernatant was removed and fresh N2 media plus 1μM Dorsomorphin was added. EBs were aliquoted to a new ultra-low attachment plate. From this point, EBs were fed every 2 days until day 10 when neuronal differentiation was induced.

**Neuronal Differentiation (Protocol B)**

Neurons were derived from neural progenitor cells (NPCs) as described by Marchetto et al. with slight modifications (4). On day 10, EBs were collected in a 15ml tube and allowed to settle by gravity for 5 minutes. Supernatant was removed and EBs were resuspended gently in NBF medium (DMEM/F12, 0.5X N2, 0.5X B27, 1% p/s) plus fresh 20ng/ml FGF2 (R&D Systems). Carefully with a 10ml pipet, EBs were aliquoted to a matrigel (BD Biosciences) plate. On day 12, plates were checked for rosette formation and fed with NBF medium plus fresh FGF2. Rosettes were fed every other day.

Rosettes were carefully excised on day 16 with a 26g needle and pooled in a 1.5ml tube. Accutase (ICT) was added to the rosettes for 3 minutes at 37oC. After incubation, rosettes were broken up with a 1ml pipet tip, centrifuged for 2 minutes at 1,000rpm, and washed once with 1X PBS (Invitrogen). The pellet was resuspended as to single cells in NBF media with fresh FGF2 (20ng/ml) and aliquoted to Poly-L-Ornithine (Sigma)/Laminin (Roche) plates. NPCs were fed every other day.

Once NPCs reached 40-50% confluence, neural differentiation was induced by withdrawing FGF2. NBF media was supplemented with fresh growth factors as follows: WNT3A (100ng/ml) (R&D Systems), BDNF (10ng/ml), GDNF (10ng/ml), IGF-1 (10ng/ml) (PeproTech), and cAMP 1μM (Sigma). Cells were fed every other day.

Protocol “B” produces a more heterogenous mix of glutamatergic neurons and GABAergic neurons in ~50-50 to 60-40 ratio based on immunocytochemistry (not shown).

**RNA-Seq**

Total RNA was isolated from cells using the miRNeasy Kit (Qiagen) according to the manufacturer’s protocol. A DNase1 digestion step was included. Total cellular rather than polyA+ RNA was used so that intronic sequences could be captured, since these are enriched for informative SNPs compared with coding regions. Briefly, total RNA (25 ng) was converted to cDNA using the NuGEN Ovation RNA-Seq System according to the manufacturer’s protocol (NuGEN, San Carlos, CA, USA). The protocol employs a single primer isothermal amplification (SPIA) method to amplify RNA target into double stranded cDNA under standardized conditions that markedly deplete rRNA without preselecting mRNA. cDNA was then used for Illumina sequencing library preparation using Encore NGS Library System I. NuGEN-amplified double-stranded cDNA was fragmented into ~300 base pair (bp) using a Covaris-S2 system. DNA fragments (200 ng) were then end-repaired to generate blunt ends and adapters were ligated for paired end sequencing on Illumina HiSeq 2000. The purified cDNA library products were evaluated using the Agilent bioanalyzer and diluted to 10 nM for cluster generation in situ on the HiSeq paired-end flow cell using the CBot automated cluster generation system followed by massively-parallel sequencing (2x100 bp). We obtained 104 bp mate-paired reads from DNA fragments of average length of 250-bp (standard deviation for the distribution of inner distances between mate pairs is approximately 50 bp). iPSC and neuron RNA-Seq reads were separately aligned to the human genome (GRCh37/hg19) using the software TopHat (version 1.1.4) (5).

Splice junctions were automatically determined by TopHat, with the provided guidance of annotated gene models (GTF file) obtained mainly from Ensembl (http://www.ensembl.org). The resulting alignment data from Tophat were then fed to Cufflinks (version 0.9.3) to assemble aligned RNA-Seq reads into transcripts [29]. Annotated transcripts were obtained from the UCSC genome browser (http://genome.ucsc.edu) and the Ensembl database; the category of transcripts was described at http://vega.sanger.ac.uk/info/about/gene_and_transcript_types.html. Transcript abundances were measured in Fragments Per Kilobase of exon per Million fragments mapped (FPKM) (6). To address the common issue in the assembly that a read may align to multiple isoforms of the same gene or multiple transcripts within the same genetic locus, maximum likelihood estimation was performed by Cufflinks based on a numerical optimization algorithm for calculating FPKM. Finally, the program Cuffdiff was used to define differential expression 5. A comprehensive description of the fundamental findings was previously reported (3).

**RT-PCR and DNA sequencing**

Total RNA was extracted using a PicoPure® RNA Isolation Kit according to the manufacturer’s instructions (Arcturus Bioscience/Molecular devices, Sunnyvale, CA). An additional treatment with DNase1 (Qiagen, Valencia, CA) was included to remove genomic DNA. Reverse transcribed PCR (RT-PCR) was performed using a OneStep RT-PCR Kit (Qiagen, Valencia, CA) according to the manufacturer’s instructions. Samples were purified using QIAquick (Qiagen) to remove unused primers. Sanger dideoxy sequencing was carried out with fluorescent nucleotides using ABI 3730 sequencer. Either the forward or reverse primer used for RT-PCR amplification was used for sequencing, depending on the quality of test sequencing.

**Inducing iPSCs To Differentiate Into 3 Germ Layers**

iPSCs were washed once with DPBS, 2 ml per well. Then, 0.5 ml of CTK solution (CTK Solution (500μl 2.5% trypsin [25 mg in 1 ml, 1 mM HCl], 500 μl Collagenase IV [1 mg/ml working stock solution], 50 μl 0.1M CaCl2, 1 ml KO serum replacement, 3 mls H_2_O) was added and the cells were incubated for 5 min at 37^o^C. The wells were then washed once with 2 mls DPBS without CTK (note that CTK is harsh on cells and causes iPSC colonies to detach easily, care must be taken not to aspirate iPSC colonies during washes). Then 2 mls hES medium was added to the wells and remaining iPSC colonies were detached with a cell scraper, with care taken to avoid breaking up colonies. The colonies were then transferred to a 15 ml tube, and the colonies were allowed to settle to bottom of tube for 5 min. The supernatant was carefully removed and fresh hES medium without FGF2 was added. The colonies were aliquoted with a 10 ml pipet to 6-well low-attachment cluster plates (2 mls per well). Plates were incubated for 2 days at 37^o^C, 5% CO2. Medium was changed as in first section of methods. Thereafter, medium was changed every 2 days.

On day 8, chamber slides coated with 0.1% matrigel were prepared. The embryoid bodies were transferred to the chamber slides containing 0.5ml hES and fed every other day by removing medium and adding fresh hES. After 8 additional days, immunocytochemistry for differentiated cell markers was carried out.

**Immunocytochemistry**

Immunocytochemistry was carried as previously described (7, 8). A list of the antibodies used in the study is shown in below.

**Primers used for to amplify samples for sequencing**

NRG1; rs1481757

F-AGGGGAGAATGGAAGGAGAA

R-CCAGCACACAAAAGAGCAAA

179bp

NRG1; rs4602844

F-CATTTGCCTGGGTTTGTTTT

R-TGTGGTGGTGTGTTATGCAA

249bp

ERBB4; rs10175279

F-TCCAAAGAAGGAGCCGTAGA

R-GCACAACTGCATGAAGCATTA

157bp

NPAS3; rs10483449

F-TTTGCAGAAACGCATCAGAC

R-CCGAAAGGGATCAAAAGTGA

A2BP1 (RBFOX1); rs17139246

F-TCCCCAAGGTGTTTGTCAGT

R-TGCTTACCAGATTCCAAAACC

172 bp

A2BP1; rs8051129/rs8051142

F-TGATGTTAGCAAACCCTGTCA

R-TGTTGGGAACATCAAGAGCA

164 bp

A2BP1; rs8051751

F-ATTGCTGAGAGGGTGCAGAT

R-CTGGTGCTTTGAGCATCCTT

216

A2BP1; rs1319358

F-TGGCTTCCAGGACCTCTAAA

R-TGGGAGGTTACAAGGGAAGA

194bp

NPAS3; rs10454679

F-CCCAAGCATTATCAATTGCAG

R-CAGGTTGAAGGGGAGGAAGT

178

AUTS2; rs17141606

F-TGCTTGGTATCAAAAGGATGG

R-GGCAGCAGAGAAGGAGAGAA

177bp

TSPAN6; rs8575

F-GGGATACAGGGTTTCAACGA

R-AAAGTTGCTTGGCTGGAGAA

226 bp

NXT2; rs3204027

F-TTGGAGTCAAAGCCATTGTT

R-GCAGTTGACAGTACAAAGCAC

150bp

KAL1; rs5978914

F-CATTGCATTTGGCTATTTGG

R-TGGTGATGGTGGTGGTTATG

CTNNA3; rs1925621; rs1925622

F-TTCCTTTTCCCAGGTCACAC

R-TTGAATCCCTAAAGCCTCCA

229 bp

GABRA3; rs66277588

F-TGTCCCCTTTCCCTGATGTA

R-TGTGTTCGTGGAAAGCAAAG

182bp

KCNQ1; rs462402

F-CGTGGGGTTCTTAAACAGGA

R-TGTTGTTCCCTCCTCAGCTT

246bp

KCNQ1; rs463924

F-TTTGAAAAGGAGAGCAGAGGA

R-GCTGGCTTTCCAACATCATC

195bp

**Antibodies used in developing iPSCs**

1^o^ Antibody 2^o^ Antibody

Anti-OCT-4 [POU5F1] Millipore cat#MAB4419 DyLight 488-conjugated AffiniPure Goat Anti-Mouse IgG (H+L) Jackson Immuno Research cat#115-485-003

Phycoerythrin (PE) anti-human TRA-1-60 N/A

eBioscience cat#12-8863

Phycoerythrin (PE) anti-human TRA-1-81 N/A

eBioscience cat#12-8883

Alexa Fluor 488 anti-mouse/human SSEA-3 Alexa Fluor 488 goat anti-rat IgM (μ chain)

eBioscience cat#53-8833 Invitrogen cat#A21212

FITC Mouse anti-SSEA-4 BD Biosciences Alexa Fluor 488 rabbit anti-mouse IgG

Cat# 560126 (H+L) Invitrogen cat#A11059

Anti-human/mouse α-Fetoprotein Alexa Fluor 488 rabbit anti-mouse IgG

R&D Systems cat#MAB1368 (H+L) Invitrogen cat#A11059

Anti-Desmin (Muscle Cell Marker) Ab-1 Alexa Fluor 488 rabbit anti-mouse (clone D33) IgG Thermo Scientific cat#MS-376-S1 (H+L) Invitrogen cat#A11059

anti-Synapsin (rabbit polyclonal, 1:1000

Chemicon/MIllipore, Temecula, CA) Donkey anti-rabbit Jackson Immunoresearch (JI), West Grove, PA).

anti-VGLUT2 (mouse monoclonal, 1:1000 Donkey anti-mouse, JI

Synaptic Systems Goettingen, Germany),

cat#135403

anti-MAP2 (mouse monoclonal, 1:1000, Donkey anti-mouse, JI

Chemicon.

anti GABA (mouse or rabbit, 1:5000 Donkey anti-mouse, anti rabbit, JI

Sigma Aldrich, St. Louis, MO).

Sheep/Tyrosine Hydroxylase Pel-Freez

Cat#P60101

anti-Synapsin (rabbit polyclonal, 1:1000 Donkey anti rabbit, JI

Chemicon/MIllipore, Temecula, CA)

All secondary Ab labeld with Cy3, FITC, or Cy5, Jackson Immunoresearch, West Grove, PA.

DAPI nuclear stain: ProLong Gold antifade reagent with DAPI, Invitrogen cat#P-36931

References

1. Takahashi K, Tanabe K, Ohnuki M, Narita M, Ichisaka T, Tomoda K, et al. Induction of pluripotent stem cells from adult human fibroblasts by defined factors. Cell 2007; **131:** 861-72.

2. Pedrosa E, Sandler V, Shah A, Carroll R, Chang C, Rockowitz S, et al. Development of patient-specific neurons in schizophrenia using induced pluripotent stem cells. J Neurogenet 2011; .

3. Lin M, Pedrosa E, Shah A, Hrabovsky A, Maqbool S, Zheng D, et al. RNA-seq of human neurons derived from iPS cells reveals candidate long non-coding RNAs involved in neurogenesis and neuropsychiatric disorders. PLoS One 2011; **6:** e23356.

4. Marchetto MC, Carromeu C, Acab A, Yu D, Yeo GW, Mu Y, et al. A model for neural development and treatment of rett syndrome using human induced pluripotent stem cells. Cell 2010; **143:** 527-39.

5. Trapnell C, Pachter L, Salzberg SL. TopHat: Discovering splice junctions with RNA-seq. Bioinformatics 2009; **25:** 1105-11.

6. Mortazavi A, Williams BA, McCue K, Schaeffer L, Wold B. Mapping and quantifying mammalian transcriptomes by RNA-seq. Nat Methods 2008; **5:** 621-8.

7. Davidkova G, Carroll RC. Characterization of the role of microtubule-associated protein 1B in metabotropic glutamate receptor-mediated endocytosis of AMPA receptors in hippocampus. J Neurosci 2007; **27:** 13273-8.

8. Huangfu D, Osafune K, Maehr R, Guo W, Eijkelenboom A, Chen S, et al. Induction of pluripotent stem cells from primary human fibroblasts with only Oct4 and Sox2. Nat Biotechnol 2008; **26:** 1269-75.
